# Changes in Children’s Oral Health Status and Receipt of Preventive Dental Visits, United States, 2003–2011/2012

**DOI:** 10.5888/pcd10.130187

**Published:** 2013-12-05

**Authors:** Mahua Mandal, Burton L. Edelstein, Sai Ma, Cynthia S. Minkovitz

**Affiliations:** Author Affiliations: Mahua Mandel, Burton L. Edelstein, Sai Ma; College of Dental Medicine and Mailman School of Public Health, Columbia University, New York, New York.

## Abstract

**Introduction:**

Oral health represents the largest unmet health care need for children, and geographic variations in children’s receipt of oral health services have been noted. However, children’s oral health outcomes have not been systematically evaluated over time and across states. This study examined changes in parent-reported children’s oral health status and receipt of preventive dental visits in 50 states and the District of Columbia.

**Methods:**

We used data from the 2003 and the 2011/2012 National Survey of Children’s Health. National and state-level estimates of the adjusted prevalence of oral health status and preventive dental visits were calculated and changes over time examined. Multivariable logistic regression was used to compare outcomes across all states and the District of Columbia for each survey year.

**Results:**

The percentage of parents who reported that their children had excellent or very good oral health increased from 67.7% in 2003 to 71.9% in 2011/2012. Parents who reported that their children had preventive dental visits increased from 71.5% in 2003 to 77.0% in 2011/2012. The prevalence of children with excellent or very good oral health status increased in 26 states, and the prevalence of children who received at least 1 preventive care dental visit increased in 45 states. In both years, there was more variation among states for preventive dental visits than for oral health status.

**Conclusions:**

State variation in oral health status and receipt of preventive dental services remained after adjusting for demographic characteristics. Understanding these differences is critical to addressing the most common chronic disease of childhood and achieving the oral health objectives of Healthy People 2020.

## Introduction

Oral health is critically important to overall health (1) and is the largest unmet health care need for children (2). Dental caries, a primary indicator of oral health, remains the most common chronic childhood disease (3). Forty percent of children aged 2 to 11 years experience dental caries (4), and its prevalence is increasing in the youngest age group: among children aged 2 to 4 years, the prevalence of children with dental caries increased from 19% in 1988–1994 to 24% in 1999–2004 (5). Moreover, parents report their children’s oral health status as worse than their general health status (6).

Children do not receive dental preventive care in accordance with the recommendations of professional organizations. The American Academy of Pediatric Dentistry and the American Academy of Pediatrics recommend that children visit a dentist every 6 months beginning by age 12 months (7,8); however, according to the 2007 National Survey of Children’s Health, 22% of children under age 17 did not visit a dentist in the previous year (9). Furthermore, although 1 of the goals of Healthy People 2010 was that 66% of low-income children and adolescents receive annual preventive-care dental services, data from Medicaid’s Early and Periodic Screening, Diagnostic, and Treatment program indicate only 36% of Medicaid-eligible children under 19 years received any dental preventive care in fiscal year 2010 (10). In 2003 and 2007, publicly insured and uninsured children had lower odds of making preventive care visits to a dentist and higher odds of experiencing delayed dental care than privately insured children (11). Finally, compared with white, black, and Hispanic children and children of other races/ethnicities are less likely to receive oral health care, including preventive dental visits (11,12), and more likely to experience delayed care or have unmet dental care needs (13).

Although geographic variations exist in children’s receipt of oral health care (14), oral health status and receipt of preventive dental care by children have not been systematically evaluated over time and across states. Assessing state-level variations in children’s oral health over time is necessary for understanding observed improvements or worsening of outcomes and the potential role of policies and programs in influencing these changes. The objective of this study was to examine changes in parent-reported children’s oral health status and recent preventive dental visits in the United States over time and across 50 states and the District of Columbia.

## Methods

### Data source and sample

We analyzed data from the 2003 National Survey of Children’s Health (NSCH) and the 2011/2012 NSCH. These surveys were conducted by the Centers for Disease Control and Prevention’s National Center for Health Statistics and sponsored by the Maternal and Child Health Bureau, Health Resources and Services Administration. The NSCH is a telephone survey of parents or caregivers that measures health and health care use in a nationally representative sample of noninstitutionalized children aged 0 to 17 years in the United States (15,16). The 2003 survey was administered in English and Spanish, and the 2011/2012 survey was administered in English, Spanish, Korean, Vietnamese, Mandarin, and Cantonese. We included children aged 1 through 17 years for whom complete data on oral health status or preventive dental visits were available. Our final sample sizes were 96,510 for 2003 (94.3% of total sample) and 90,555 for 2011/2012 (94.6% of total sample) (15,16).

### Study measures

To determine oral health status, both the 2003 and 2011/2012 surveys asked respondents to describe the condition of their children’s teeth as excellent, very good, good, fair, or poor. Responses were dichotomized into excellent/very good and good/fair/poor.

The 2003 survey asked respondents if their children had visited a dentist in the past 12 months for any routine preventive care including check-ups, screenings, and sealants; the question was directed at respondents who reported their children had visited any type of dentist in the past year. The 2011/2012 survey asked respondents how many times their children had visited a dentist in the past 12 months for preventive dental care, including check-ups and dental cleanings; the question was asked among those reporting their children had visited a dentist for any kind of dental care in the past year. A dichotomous variable was created for each survey year indicating children who received at least 1 recent preventive dental visit and children who did not.

Previous research identifying child and family characteristics associated with children’s oral health informed the selection of independent variables for this study (9,13). Child characteristics included age (1–2 y,3–5 y,6–11 y,12–17 y); sex; race (non-Hispanic white, non-Hispanic black, Hispanic, other or multiracial); overall health status at time of survey (good/fair/poor, excellent/very good); health insurance (none, Medicaid or Children’s Health Insurance Program, private or other); and number of times child had moved in his or her lifetime (none, 1–2, 3–4, 5 or more times). Family characteristics included primary language spoken at home (English, other); highest level of education in household (less than high school graduate, high school graduate, more than high school graduate); number of children aged less than 18 years living in the household (1, 2, 3, 4 or more); number of adults living in household (1, 2, 3 or more); and household poverty level (≤100% federal poverty level [FPL]; 101%–200% FPL; 201%–300% FPL; 301%–400% FPL; >400% FPL).

### Missing data

Missingness was examined for each variable. Household poverty level had about 8% missing data for each year; multiple imputation techniques were employed that used 5 imputed values provided by the NSCH (15,16). Missing data for the remaining variables, which did not exceed 2.2%, were recoded as part of the reference group. Sensitivity analysis excluding missing data yielded similar results to analysis conducted with recoded missing data.

### Statistical analysis

All analyses used weighted data and accounted for the clustered design of the NSCH. First, bivariate and multivariable logistic regressions were used to examine the unadjusted and adjusted relations, respectively, between child and family characteristics and each dependent variable, oral health status, and preventive dental visits. Average marginal effects (17) were calculated to estimate the adjusted prevalence (adjusting for child and family characteristics) of oral health status and preventive dental visits in the United States and for each of the 50 states and the District of Columbia, in 2003 and 2011/2012. Significance of the change over time in estimated adjusted prevalence was determined by performing contrast testing (18) between 2011/2012 and 2003 outcomes. Subgroup analyses for the full samples were performed by children’s insurance status and household poverty level. Finally, to examine state-level variation, multivariable logistic regression was used to compare the adjusted odds of each dependent variable across the 50 states and the District of Columbia. As the reference category, we used Nevada, the state with the lowest adjusted prevalence of children with excellent or very good oral health status in both survey years, and the lowest and second lowest adjusted prevalence of children having at least 1 recent preventive dental visit in 2003 and 2011/2012, respectively.

## Results

### Oral health status and preventive dental visit and child and family characteristics, 2011/2012

In unadjusted analyses, having excellent or very good oral health status and at least 1 preventive dental visit in the past 12 months in 2011/2012 was associated with children who were white, had health insurance, had excellent or very good health status, had never moved, lived in families that spoke primarily English, lived with an adult who graduated high school, and lived in families whose income exceeded the FPL. Additionally, children had higher odds of having excellent or very good oral health status if they were aged 1 to 2 years (compared with being aged 3–17 years); female; lived with no other children or 1 other child; and lived with 2 adults. In contrast, children had higher odds of having at least 1 preventive visit if they were older and lived with other children.

Except for children who had never moved, individual characteristics associated with excellent or very good oral health status in the unadjusted analysis remained significant in adjusted analysis (Table 1). Moving fewer than 5 times was associated with excellent or very good oral health status. Family characteristics associated with more favorable oral health status included living with an adult who had more than high school education and having an income more than 200% above the FPL. There were some differences between the adjusted and unadjusted associations for children receiving at least 1 preventive dental visit. Only Hispanic children were less likely than white children to receive at least 1 preventive dental visit, as were children who moved 5 times or more. Similar to the adjusted associations for oral health status, children were more likely to have a preventive dental visit if they lived with someone who had more than high school education and in a household with an income more than 200% above the FPL. Additionally, children who lived with other children were more likely to have had a preventive dental visit.

**Table 1:** Adjusted Odds of Parent-Reported Child’s Oral Health Status and Receipt of Preventive Dental Visit in the United States, by Child and Family Characteristics^a^, 2011/2012

Child Characteristics	Oral Health Status Reported as Excellent or Very Good	At Least 1 Preventive Dental Visit in 12 Months Preceding Survey
	Adjusted Odds Ratio (*P* Value)	Adjusted Odds Ratio (*P* Value)
**Child Characteristics**		
**Age, y**		
1–2	1 [Reference]	
3–5	0.47 (<.001)	8.6 (<.001)
6–11	0.28 (<.001)	25.3 (<.001)
12–17	0.33 (<.001)	21.4 (<.001)
**Sex**		
Male	1 [Reference]	
Female	1.17 (<.001)	1.06 (.20)
**Race/ethnicity**		
Non-Hispanic white	1 [Reference]	
Non-Hispanic black	0.76 (<.001)	0.95 (.40)
Hispanic	0.76 (<.001)	0.76 (<.001)
Multiracial or other race	0.83 (.005)	1.12 (.17)
**Health insurance**		
None	1 [Reference]	
Medicaid or Children’s Health Insurance Program	1.25 (.01)	3.70 (<.001)
Private or Other	1.59 (<.001)	3.43 (<.001)
**Overall health status**		
Good, fair, or poor	1 [Reference]	
Excellent or very good	4.73 (<.001)	1.17 (.02)
**No. times child has moved**		
None	1 [Reference]	
1–2	1.01 (.77)	1.06 (.28)
3–4	0.90 (.06)	0.97 (.69)
≥5	0.81 (.001)	0.79 (.004)
**Family Characteristics**		
**Primary language spoken at home**		
Not English	1 [Reference]	
English	1.83 (.19)	0.86 (.13)
**No. children aged under 18 years living in household**		
1	1 [Reference]	
2	1.05 (.27)	1.42 (<.001)
3	0.98 (.67)	1.58 (<.001)
≥4	1.08 (.27)	1.57 (<.001)
**No. adults living in household**		
1	1 [Reference]	
2	0.92 (.20)	1.01 (.87)
≥3	0.91 (.15)	0.88 (.11)
**Highest level of education attained by anyone in household**		
Less than high school graduate	1 [Reference]	
High school graduate	1.31 (<.001)	1.23 (.02)
More than high school graduate	1.76 (<.001)	1.53 (<.001)
**Household income level, percentage of federal poverty level**		
≤100%	1 [Reference]	
101%–200%	1.09 (.17)	1.12 (.10)
201%–300%	1.37 (<.001)	1.31 (.004)
301%–400%	1.61 (<.001)	1.81 (<.001)
>400%	1.96 (<.001)	2.31 (<.001)

a Based on multivariable model adjusting for age, sex, race/ethnicity, health insurance, overall health status, number of times child moved, primary language spoken at home, total number of children and adults in household, highest level of education in household, and household income percentage of federal poverty level).

### Adjusted prevalence of oral health status and preventive dental care between 2003 and 2011/2012

The adjusted prevalence of children’s oral health status reported as excellent or very good significantly increased in the United States, from 67.7% in 2003 to 71.9% in 2011/2012 (*P* < .001). Although prevalence of excellent or very good oral health status did not increase among children without health insurance, it did increase among children with public health insurance (55.6% to 59.4%, *P* < .001) and private health insurance (76.4% to 81.1%, *P* < .001). Favorable oral health status increased for children at all household poverty levels. The prevalence of children with excellent or very good oral health status living at or below the FPL increased from 48.9% to 52.4% (*P* = .001) and for those living more than 400% above the FPL, it increased from 82.5% to 86.3% (*P* < .001).

Significant improvements in parent reports of children’s oral health status were observed among 26 states (Table 2). Parents in Utah reported the most improvement (69.1% to 79.2%, *P* < .001), and those in Missouri reported the least improvement that was significant (71.4% to 75.0%, *P* < .001).

**Table 2:** Adjusted Prevalence and Percentage Point Change of Children’s Oral Health Status and Children’s Receipt of at Least 1 Preventive Dental Visit, 50 States and the District of Columbia^a^, 2003 and 2011/2012 National Survey of Children’s Health in 12 Months Preceding Survey

State or District of Columbia	Oral Health Status Reported as Excellent or Very Good			At Least 1 Preventive Dental Visit In 12 Months Preceding Survey		
	2003^b^ Adjusted Prevalence (95% CI)	2011/2012^b^ Adjusted Prevalence (95% CI)	Percentage Point Change (*P* Value^c^	2003^b^ Adjusted Prevalence (95% CI)	2011/2012^b^ Adjusted Prevalence (95% CI)	Percentage Point Change (*P* Value)^c^
United States, total	67.7 (67.2–68.2)	71.9 (71.3–72.5)	4.2 (<.001)	71.5 (71.0–72.0)	77.0 (76.4–77.5)	5.5 (<.001)
Alaska	71.5 (69.1–74.0)	73.0 (70.0–76.0)	1.5 (.364)	74.2 (72.1–76.4)	71.7 (69.0–74.5)	−2.5 (.342)
Alabama	68.1 (65.7–70.5)	71.5 (68.5–74.4)	3.4 (.069)	72.8 (70.6–75.0)	79.2 (76.6–81.8)	6.4 (<.001)
Arkansas	65.1 (62.5–67.6)	70.4 (67.5–73.2)	5.3 (.003)	67.5 (65.0–70.0)	73.5 (70.7–76.4)	6.0 (<.001)
Arizona	61.2 (58.8–63.6)	66.4 (63.5–69.2)	5.2 (.007)	66.1 (63.7–68.4)	75.1 (72.6–77.7)	9.0 (<.001)
California	58.5 (57.3–60.8)	65.1 (62.3–68.0)	6.6 (.001)	70.3 (68.2–72.5)	75.1 (72.4–77.8)	4.8 (.008)
Colorado	69.0 (66.7–71.3)	71.1 (68.4–73.9)	2.1 (.216)	70.9 (68.8–73.1)	76.6 (73.7–79.5)	5.7 (.001)
Connecticut	75.2 (73.1–77.4)	77.5 (75.2–79.8)	2.3 (.306)	79.8 (78.0–81.7)	86.0 (83.9–88.1)	6.2 (<.001)
District of Columbia	66.1 (64.0–68.6)	72.7 (69.2–76.2)	6.6 (.002)	70.0 (67.6–72.2)	82.7 (79.9–85.5)	12.7 (<.001)
Delaware	69.8 (67.5–72.0)	73.8 (70.9–76.7)	4.0 (.060)	71.6 (69.6–73.6)	77.3 (74.8–79.7)	5.7 (<.001)
Florida	68.1 (65.7–70.5)	70.4 (67.7–73.1)	2.3 (.279)	64.2 (61.8–66.6)	67.3 (64.5–70.0)	3.1 (.060)
Georgia	69.1 (66.5–71.7)	74.6 (71.9–77.3)	5.5 (.002)	71.5 (69.2–73.8)	75.7 (73.1–78.3)	4.2 (.012)
Hawaii	70.6 (68.1–73.1)	73.2 (70.6–75.8)	2.6 (.122)	77.4 (75.3–79.6)	83.1 (80.8–85.2)	5.7 (<.001)
Iowa	72.5 (70.2–74.7)	73.2 (70.7–76.8)	0.7 (.955)	76.8 (74.9–78.8)	82.6 (80.5–84.8)	5.8 (<.001)
Idaho	69.2 (66.9–71.6)	72.0 (69.0–75.0)	2.8 (.154)	72.0 (69.9–74.1)	78.3 (75.7–81.0)	6.3 (<.001)
Illinois	66.8 (64.4–69.1)	73.6 (71.1–76.2)	6.8 (<.001)	72.3 (70.2–74.5)	80.3 (78.0–82.7)	8.0 (<.001)
Indiana	72.3 (69.9–74.6)	72.6 (69.8–75.5)	0.3 (.477)	73.8 (71.6–76.0)	78.3 (75.8–80.7)	4.5 (.003)
Kansas	71.3 (68.8–73.7)	73.0 (70.3–75.6)	1.7 (.332)	73.3 (71.1–75.5)	79.0 (76.7–81.4)	5.7 (<.001)
Kentucky	70.9 (68.5–73.3)	75.8 (73.1–78.5)	4.9 (.008)	72.4 (70.1–74.7)	75.8 (73.2–78.4)	3.4 (.016)
Louisiana	68.6 (66.2–71.0)	70.3 (67.3–73.3)	1.7 (.348)	67.5 (65.2–69.9)	78.9 (76.3–81.5)	11.4 (<.001)
Massachusetts	72.7 (70.5–74.9)	79.4 (77.0–81.9)	6.7 (<.001)	79.0 (77.4–80.7)	82.9 (80.9–85.0)	3.9 (.001)
Maryland	73.9 (71.7–76.0)	75.9 (73.1–78.7)	2.0 (.169)	72.8 (70.8–74.8)	79.0 (76.4–81.6)	6.2 (<.001)
Maine	78.7 (76.5–80.8)	79.9 (77.5–82.4)	1.2 (.413)	76.3 (74.4–78.1)	80.7 (78.4–83.1)	4.4 (.001)
Michigan	71.4 (69.1–73.6)	76.7 (74.1–79.3)	5.3 (.003)	74.4 (72.4–76.3)	78.2 (75.9–80.6)	3.8 (.004)
Minnesota	73.1 (70.7–75.5)	79.1 (76.7–81.5)	6.0 (.001)	75.2 (73.2–77.3)	77.0 (74.7–79.2)	1.8 (.188)
Missouri	71.4 (69.2–73.6)	75.0 (72.3–77.6)	3.6 (.045)	68.3 (66.2–70.3)	72.9 (70.3–75.5)	4.6 (.006)
Mississippi	61.5 (58.9–64.1)	70.9 (67.8–74.1)	9.4 (<.001)	66.5 (64.1–69.0)	73.9 (71.0–76.8)	7.4 (<.001)
Montana	71.1 (68.7–73.4)	73.5 (70.7–76.2)	2.4 (.217)	70.5 (68.3–72.8)	76.2 (73.6–78.9)	5.7 (<.001)
North Carolina	69.0 (66.6–71.4)	75.5 (72.7–78.2)	6.5 (.002)	71.2 (69.1–73.4)	75.7 (72.9–78.5)	4.5 (.002)
North Dakota	74.0 (71.7–76.4)	79.7 (77.1–82.4)	5.7 (.002)	73.7 (71.5–75.9)	75.4 (72.8–77.9)	1.7 (.333)
Nebraska	73.8 (71.4–76.1)	73.2 (70.7–75.7)	−0.6 (.970)	75.6 (73.6–77.5)	79.8 (77.4–82.2)	4.2 (.005)
New Hampshire	78.1 (76.1–80.2)	82.2 (79.9–84.5)	4.1 (.010)	79.9 (78.2–81.6)	84.9 (82.9–86.9)	5.0 (<.001)
New Jersey	70.5 (68.3–72.7)	74.5 (71.9–77.2)	4.0 (.078)	73.1 (71.1–75.2)	79.3 (77.0–81.6)	6.2 (<.001)
New Mexico	62.5 (59.8–65.2)	63.7 (60.5–66.9)	1.2 (.660)	70.1 (67.5–72.6)	80.5 (77.7–83.3)	10.4 (<.001)
Nevada	58.3 (56.0–60.7)	62.9 (60.0–65.8)	4.6 (.017)	63.6 (61.4–65.8)	67.4 (64.3–70.4)	3.8 (.002)
New York	69.2 (66.9–71.5)	70.8 (68.3–73.4)	1.6 (.337)	73.7 (71.5–75.8)	77.4 (75.2–79.6)	3.7 (.009)
Ohio	71.9 (69.7–74.2)	74.6 (71.8–77.4)	2.7 (.052)	73.7 (71.8–75.7)	77.7 (75.3–80.0)	4.0 (.008)
Oklahoma	67.9 (65.5–70.3)	72.8 (70.1–75.6)	4.9 (.006)	64.6 (62.3–67.0)	72.9 (70.4–75.4)	8.3 (<.001)
Oregon	67.5 (65.2–69.8)	71.4 (68.8–74.0)	3.9 (.012)	69.8 (67.7–72.0)	76.6 (74.2–79.0)	6.8 (<.001)
Pennsylvania	72.2 (70.0–74.5)	75.1 (72.2–77.9)	2.9 (.146)	75.1 (73.3–77.0)	79.6 (77.2–82.0)	4.5 (.002)
Rhode Island	71.3 (68.9–73.6)	76.8 (74.2–79.4)	5.5 (.001)	77.6 (75.7–79.5)	82.2 (80.1–84.3)	4.6 (.001)
South Carolina	68.3 (66.0–70.6)	76.0 (73.4–78.7)	7.7 (<.001)	70.9 (68.7–73.0)	77.2 (74.5–80.0)	6.3 (<.001)
South Dakota	75.9 (73.5–78.3)	78.2 (75.6–80.8)	2.3 (.190)	70.1 (67.8–72.4)	77.7 (75.4–79.9)	7.6 (<.001)
Tennessee	67.7 (65.2–70.2)	72.6 (69.9–75.4)	4.9 (.002)	70.8 (68.4–73.1)	78.4 (75.8–81.0)	7.6 (<.001)
Texas	61.5 (59.2–63.8)	66.1 (63.0–69.1)	4.6 (.023)	66.7 (64.5–69.0)	76.4 (73.4–79.4)	9.7 (<.001)
Utah	69.1 (66.5–71.7)	79.2 (76.9–81.6)	10.1 (<.001)	73.4 (71.2–75.7)	77.4 (75.2–79.5)	4.0 (.008)
Virginia	75.0 (72.8–77.2)	79.6 (76.9–82.2)	4.6 (.012)	72.4 (70.5–74.4)	78.9 (76.3–81.5)	6.5 (<.001)
Vermont	79.3 (77.1–81.4)	83.3 (81.0–85.6)	4.0 (.010)	84.6 (83.0–86.2)	88.1 (86.3–89.9)	3.5 (.003)
Washington	69.6 (67.2–72.0)	71.7 (69.0–74.4)	2.1 (.260)	75.5 (73.4–77.5)	85.5 (83.2–87.7)	10.0 (<.001)
Wisconsin	74.1 (71.8–76.5)	77.0 (74.5–79.6)	2.9 (.080)	77.8 (75.8–79.8)	79.1 (77.1–81.2)	1.3 (.326)
West Virginia	74.6 (72.4–76.8)	76.5 (74.0–79.0)	1.9 (.116)	74.5 (72.6–76.5)	80.4 (78.3–82.6)	5.9 (<.001)
Wyoming	69.5 (67.2–71.8)	75.8 (73.3–78.4)	6.3 (.001)	76.2 (74.3–78.1)	77.6 (75.3–80.0)	1.4 (.188)

Abbreviation: CI, confidence interval.

a Based on multivariable model adjusting for age, sex, race/ethnicity, health insurance, overall health status, number of times child moved, primary language spoken at home, total number of children and adults in household, highest level of education in household, and household income (percentage of federal poverty level).

b Average marginal effects used to estimate adjusted prevalences and confidence intervals.

c Contrast testing used to assess significance of changes over time.

The adjusted prevalence of children who received at least 1 preventive dental visit in the past 12 months also significantly increased in the United States, from 71.5% in 2003 to 77.0% in 2011/2012 (*P* < .001). This outcome improved among children with no health insurance (50.3% to 54.1%, *P* = .028), public health insurance (from 64.6% to 73.3%, *P* < .001), and private health insurance (from 78.0% to 81.9%, *P* < .001). Similar to reported improvements in oral health status, the prevalence of children who received at least 1 recent preventive dental visit increased at all household poverty levels (≤100% FPL, from 58.2% to 68.3%, *P* < .001; >400% FPL, from 82.3% to 85.0%, *P* < .001).

Parents in all but 6 states (Alaska, Florida, Minnesota, North Dakota, Wisconsin, and Wyoming) reported significant improvements in children receiving at least 1 preventive visit. District of Columbia had the most improvement (from 70.0% to 82.7%, *P* < .001) and Kentucky had the least among states with significant change (from 72.4% to 75.8%, *P* < .016).

### Adjusted odds of oral health status and preventive dental care, 2003 and 2011/2012

Compared with Nevada, where parents reported the worst pediatric oral health status, 39 states had higher odds of children having excellent or very good oral health in 2003 (Table 3). In the same year, parents in Maine had the highest odds of reporting positive oral health status (adjusted odds ratio [AOR], 1.74; 95% confidence interval [CI], 1.44–2.11). In 2011/2012, parents in only 19 states had higher odds of reporting excellent or very good oral health status compared with Nevada, with Vermont having the highest odds (AOR, 1.71; 95% CI, 1.31– 2.23). Compared with Nevada, in 2003, 39 states had higher odds of children receiving recent preventive dental care, and Vermont had the highest odds (AOR, 2.62; 95% CI, 2.11–3.25). In 2011/2012, all but 7 states (Alaska, California, Florida, Minnesota, Missouri, and North Dakota) had higher odds of children receiving preventive dental care, with Washington having the highest odds (AOR, 3.22; 95% CI, 2.35–4.41).

**Table 3:** Adjusted Odds of Children’s Oral Health Status Reported as Excellent or Very Good and Receipt of at Least 1 Preventive Dental Visit in 12 Months Preceding Survey, 50 States and the District Of Columbia^a^, 2003 and 2011/2012 National Survey of Children’s Health

State or District of Columbia	Oral Health Status Reported as Excellent or Very Good		At Least 1 Preventive Dental Visit in 12 Months Preceding Survey	
	2003, Adjusted Odds Ratio (95% CI)	2011/2012, Adjusted Odds Ratio (95% CI)	2003, Adjusted Odds Ratio (95% CI)	2011/2012, Adjusted Odds Ratio (95% CI)
Alaska	1.34 (1.11–1.61)	1.10 (0.85–1.42)	1.75 (1.42–2.16)	1.20 (0.92–1.58)
Alabama	1.27 (1.07–1.53)	1.20 (0.93–1.56)	1.52 (1.25–1.85)	1.96 (1.47–2.61)
Arkansas	1.15 (0.96–1.38)	1.21 (0.94–1.56)	1.15 (0.94–1.41)	1.42 (1.07–1.88)
Arizona	1.11 (0.93–1.33)	1.16 (0.90–1.49)	1.16 (0.97–1.40)	1.54 (1.17–2.02)
California	1.11 (0.93–1.32)	1.20 (0.93–1.55)	1.48 (1.22–1.80)	1.28 (0.98–1.69)
Colorado	1.20 (1.00–1.43)	1.03 (0.80–1.33)	1.22 (1.00–1.48)	1.43 (1.06–1.92)
Connecticut	1.58 (1.31–1.91)	1.40 (1.09–1.79)	1.85 (1.51–2.28)	2.58 (1.91–3.48)
District of Columbia	1.52 (1.25–1.84)	1.68 (1.25–2.26)	1.77 (1.44–2.19)	2.81 (2.00–3.94)
Delaware	1.28 (1.07–1.53)	1.19 (0.92–1.54)	1.28 (1.07–1.55)	1.42 (1.08–1.87)
Florida	1.40 (1.17–1.68)	1.18 (0.93–1.51)	0.96 (0.80–1.16)	0.85 (0.66–1.09)
Georgia	1.35 (1.11–1.62)	1.49 (1.15–1.93)	1.49 (1.22–1.83)	1.49 (1.13–1.96)
Hawaii	1.39 (1.14–1.70)	1.20 (0.93–1.54)	2.31 (1.83–2.92)	2.80 (2.07–3.79)
Iowa	1.25 (1.05–1.50)	0.93 (0.73–1.19)	1.75 (1.43–2.13)	2.23 (1.67–2.97)
Idaho	1.15 (0.96–1.37)	1.03 (0.79–1.34)	1.33 (1.10–1.61)	1.74 (1.30–2.32)
Illinois	1.18 (0.99–1.41)	1.38 (1.07–1.77)	1.36 (1.13–1.65)	1.81 (1.37–2.39)
Indiana	1.36 (1.13–1.63)	1.12 (0.87–1.45)	1.52 (1.24–1.85)	1.67 (1.26–2.22)
Kansas	1.34 (1.11–1.62)	1.10 (0.86–1.41)	1.44 (1.18–1.76)	1.72 (1.30–2.27)
Kentucky	1.31 (1.10–1.57)	1.37 (1.06–1.76)	1.45 (1.19–1.78)	1.52 (1.16–2.01)
Louisiana	1.36 (1.14–1.63)	1.19 (0.92–1.54)	1.17 (0.96–1.42)	1.82 (1.36–2.43)
Massachusetts	1.25 (1.04–1.49)	1.43 (1.10–1.85)	1.78 (1.47–2.15)	1.97 (1.48–2.62)
Maryland	1.44 (1.20–1.73)	1.26 (0.97–1.64)	1.28 (1.06–1.55)	1.54 (1.14–2.07)
Maine	1.74 (1.44–2.11)	1.46 (1.13–1.89)	1.54 (1.27–1.87)	1.79 (1.35–2.38)
Michigan	1.27 (1.07–1.52)	1.33 (1.03–1.72)	1.49 (1.24–1.81)	1.49 (1.13–1.97)
Minnesota	1.24 (1.03–1.49)	1.32 (1.02–1.70)	1.52 (1.24–1.86)	1.29 (0.99–1.69)
Missouri	1.26 (1.06–1.50)	1.14 (0.89–1.46)	1.05 (0.87–1.26)	1.10 (0.85–1.44)
Mississippi	1.03 (0.86–1.23)	1.35 (1.03–1.76)	1.22 (0.99–1.49)	1.46 (1.10–1.95)
Montana	1.20 (1.01–1.44)	0.99 (0.77–1.27	1.22 (1.01–1.47)	1.61 (1.21–2.13)
North Carolina	1.36 (1.13–1.62)	1.50 (1.15–1.96)	1.41 (1.16–1.70)	1.49 (1.12–1.98)
North Dakota	1.21 (1.01–1.45)	1.25 (0.96–1.62)	1.34 (1.10–1.63)	1.29 (0.98–1.70)
Nebraska	1.39 (1.15–1.68)	1.08 (0.84–1.37)	1.62 (1.33–1.97)	1.90 (1.43–2.54)
New Hampshire	1.46 (1.22–1.76)	1.43 (1.10–1.85)	1.68 (1.38–2.05)	2.23 (1.67–2.98)
New Jersey	1.30 (1.09–1.56)	1.24 (0.96–1.60)	1.25 (1.03–1.51)	1.49 (1.14–1.96)
New Mexico	1.28 (1.06–1.54)	1.00 (0.77–1.30)	1.38 (1.12–1.70)	2.25 (1.63–3.11)
Nevada	1 [Reference]			
New York	1.33 (1.11–1.60)	1.17 (0.92–1.50)	1.47 (1.21–1.79)	1.39 (1.07–1.79)
Ohio	1.27 (1.07–1.52)	1.18 (0.91–1.52)	1.48 (1.22–1.78)	1.55 (1.18–2.03)
Oklahoma	1.23 (1.03–1.48)	1.30 (1.01–1.67)	0.98 (0.81–1.18)	1.39 (1.07–1.81)
Oregon	1.16 (0.97–1.38)	1.12 (0.88–1.43)	1.19 (0.99–1.43)	1.50 (1.15–1.96)
Pennsylvania	1.32 (1.11–1.58)	1.18 (0.90–1.53)	1.62 (1.35–1.96)	1.69 (1.27–2.24)
Rhode Island	1.36 (1.12–1.63)	1.53 (1.18–1.99)	1.86 (1.53–2.26)	2.04 (1.55–2.69)
South Carolina	1.32 (1.10–1.57)	1.56 (1.21–2.02)	1.46 (1.20–1.78)	1.75 (1.31–2.34)
South Dakota	1.45 (1.20–1.76)	1.25 (0.96–1.61)	1.13 (0.93–1.38)	1.59 (1.21–2.08)
Tennessee	1.18 (0.99–1.42)	1.25 (0.97–1.61)	1.29 (1.06–1.58)	1.72 (1.30–2.28)
Texas	1.22 (1.03–1.45)	1.20 (0.93–1.55)	1.16 (0.97–1.40)	1.66 (1.23–2.23)
Utah	1.06 (0.87–1.28)	1.53 (1.18–1.99)	1.63 (1.33–2.01)	1.61 (1.23–2.11)
Virginia	1.53 (1.28–1.84)	1.55 (1.18–2.04)	1.38 (1.14–1.68)	1.57 (1.18–2.10)
Vermont	1.61 (1.32–1.96)	1.71 (1.31–2.23)	2.62 (2.11–3.25)	3.13 (2.30–4.24)
Washington	1.26 (1.05–1.51)	1.06 (0.83–1.36)	1.66 (1.36–2.03)	3.22 (2.35–4.41)
Wisconsin	1.41 (1.17–1.70)	1.28 (1.00–1.65)	1.88 (1.54–2.30)	1.45 (1.12–1.87)
West Virginia	1.55 (1.30–1.86)	1.47 (1.15–1.89)	1.68 (1.38–2.04)	2.13 (1.62–2.81)
Wyoming	1.05 (0.88–1.25)	1.16 (0.91–1.49)	1.60 (1.32–1.94)	1.50 (1.15–1.97)

Abbreviation: CI, confidence interval.

a Based on multivariable model adjusting for age, gender, race/ethnicity, health insurance, overall health status, number of times child moved, primary language spoken at home, total number of children and adults in household, highest level of education in household, and household income (percentage of federal poverty level).

## Discussion

Oral health status and preventive dental visits improved nationally between 2003 and 2011/2012, with greater improvement observed for preventive visits. Significant improvements were seen among children in almost all categories of health insurance and household income, although oral health status of children without health insurance did not improve over time. In both years, outcomes were better among children with health insurance than among those without and among children living in households with incomes above the FPL than in those below. These findings suggest continued disparities in children’s oral health.

Oral health status improved in half of states, and preventive dental visits improved in nearly 90% of states. No state significantly worsened in either outcome over time. Children in Nevada had the worst oral health status in 2003 and in 2011/2012. Florida had the lowest percentage, and Nevada the second lowest percentage, of children receiving at least 1 recent preventive dental visit in 2011/2012. Changes in preventive dental visits had more state-level variation than changes in oral health status. Given that estimated state prevalences were adjusted for child and family characteristics, the observed variation may be due to larger variability among programs and policies that support dental visits (eg, workforce, insurance) than among those that support health status (eg, fluoridation, school-based sealant programs). For example, a report on state pediatric dental policies found that in 2009 Medicaid paid medical staff for early preventive dental health care in 35 states, whereas only 10 states had sealant programs in place for at least 50% of schools with children at high risk of cavities (19).

Our study had several study limitations. First, estimates of children’s dental outcomes may vary depending on the survey used. For example, compared with parents surveyed in the 2003 Medical Expenditure Panel Survey (MEPS), fewer parents surveyed for the 2003 NSCH reported children aged 2 to 17 years used preventive dental care in the past 12 months (75.6% NSCH vs 78.3% MEPS). In 2007, however, this reversed, with a higher proportion of NSCH-surveyed parents reporting their children received preventive dental care (82.6% NSCH vs 80.9% MEPS). The surveys’ differing data collection methods likely contributed to differences in estimates of preventive dental care (11). NSCH asks parents about their child’s dental use in the past 12 months, whereas MEPS asks parents about the timing of the last dental visit over the course of several months or years. Additionally, MEPS interviewers use prompts to improve parents’ recall of a child’s dental visit (20,21). Although methodological discrepancies across surveys may result in overestimation or underestimation, biased estimates of changes in oral health outcomes are unlikely when using the same methods across multiple years.

A second limitation is potential response bias. Because of social desirability, parents may overestimate children’s preventive dental visits. Conversely, although parents’ recall and self-report of selected dental treatments (eg, root canal) have been found to be valid (22), they may underestimate their children’s routine dental visits (23). Given that NSCH is a representative survey, it is unlikely the direction or magnitude of response bias would change significantly across survey years. Relatedly, little research has been conducted on the validity of parents’ reports of the condition of their children’s teeth. In 1 study, compared with clinicians’ determination of restorative treatment needs, caregivers overestimated children’s positive oral health status (24). Although caregivers in our study may have overestimated children’s positive oral health status, they were asked the same question in both 2003 and 2011/2012. Furthermore, the question ascertaining preventive dental visits was slightly different in the 2 survey years. The 2003 survey asked parents to respond yes or no to whether their child had had a preventive dental visit in the past 12 months, but the 2011/2012 survey asked parents how many times in the past 12 months their child had seen a dentist for preventive care. Because the manner in which this difference may have affected changes in responses cannot be determined, we must use caution in interpreting the result. Additionally, we were not able to distinguish among various types of preventive visits or services provided (eg, dental cleaning, fluoride application, sealant). A child with 1 preventive visit may not have received all necessary preventive care.

Finally, our study includes health insurance as a covariate but does not include a measure of dental insurance. Although preventive dental care services are a covered benefit for children receiving Medicaid, our study lacks information about dental insurance for children who are covered by private health insurance. Both oral health outcomes we examined improved over time among children with private health insurance, which may indicate these children were either covered by dental insurance or that their parents paid out of pocket for dental services.

Despite these limitations, this study has numerous strengths. First, NSCH provides both national and state-level estimates, permitting analysis of geographic variation in children’s oral health outcomes. Second, children’s oral health outcomes are adjusted for an array of child and family characteristics, allowing for examination of differences beyond states’ sociodemographic variability. Finally, analyzing data from NSCH 2003 and NSCH 2011/2012 allows for sufficient passage of time between surveys to adequately assess changes. More than 75% of the increase in unadjusted prevalence of children’s oral health status and 100% of the increase in unadjusted prevalence of preventive visits took place between 2003 and 2007 (25).

National and state-level improvements in children’s oral health status and use of preventive services suggests there has been progress toward the oral health objectives of Healthy People 2020, which include reducing the proportion of children and adolescents who have dental caries and untreated dental decay and increasing the proportion of low-income children and adolescents who received any preventive dental service during the past year. Further improvements may be realized by working with primary care clinicians to ensure dental homes are established starting at an early age. Additionally, public health agencies should conduct outreach to Hispanic communities and target low-income families through the Special Supplemental Nutrition Program for Women, Infants, and Children. Geographic variability in improvements in oral health status and receipt of services suggests an ongoing need to examine programs and policies implemented at the state level, with an eye toward adapting efforts in states with the largest gains to states with the worst oral health outcomes. Understanding these differences is critical to addressing the most common chronic disease of childhood and achieving the oral health objectives of Healthy People 2020.
